# An Insulin-Modified pH-Responsive Nanopipette Based on Ion Current Rectification

**DOI:** 10.3390/s24134264

**Published:** 2024-06-30

**Authors:** Xu-Fan Wang, Yi-Fan Duan, Yue-Qian Zhu, Zi-Jing Liu, Yu-Chen Wu, Tian-Hao Liu, Ling Zhang, Jian-Feng Wei, Guo-Chang Liu

**Affiliations:** 1 Department of Histology and Embryology, School of Basic Medical Sciences, Xuzhou Medical University, Xuzhou 221004, China; 202027040207@stu.xzhmu.edu.cn (X.-F.W.); 202227040104@stu.xzhmu.edu.cn (Y.-F.D.); 202127040121@stu.xzhmu.edu.cn (Y.-Q.Z.); 202104010628@stu.xzhmu.edu.cn (Z.-J.L.); 202127040120@stu.xzhmu.edu.cn (T.-H.L.); zhanglxy@xzhmu.edu.cn (L.Z.); 2 The Second Clinical Medical College, Xuzhou Medical University, Xuzhou 221004, China; 202202020118@stu.xzhmu.edu.cn; 3 National Demonstration Center for Experimental Basic Medical Science Education, Xuzhou Medical University, Xuzhou 221004, China; 4 Jiangsu Key Laboratory of Brain Disease Bioinformation, Research Center for Biochemistry and Molecular Biology, Xuzhou Medical University, Xuzhou 221004, China

**Keywords:** nanopipettes, bovine insulin, ion current rectification, silanization and amidation, pH response

## Abstract

The properties of nanopipettes largely rely on the materials introduced onto their inner walls, which allow for a vast extension of their sensing capabilities. The challenge of simultaneously enhancing the sensitivity and selectivity of nanopipettes for pH sensing remains, hindering their practical applications. Herein, we report insulin-modified nanopipettes with excellent pH response performances, which were prepared by introducing insulin onto their inner walls via a two-step reaction involving silanization and amidation. The pH response intensity based on ion current rectification was significantly enhanced by approximately 4.29 times when utilizing insulin-modified nanopipettes compared with bare ones, demonstrating a linear response within the pH range of 2.50 to 7.80. In addition, insulin-modified nanopipettes featured good reversibility and selectivity. The modification processes were monitored using the I-V curves, and the relevant mechanisms were discussed. The effects of solution pH and insulin concentration on the modification results were investigated to achieve optimal insulin introduction. This study showed that the pH response behavior of nanopipettes can be greatly improved by introducing versatile molecules onto the inner walls, thereby contributing to the development and utilization of pH-responsive nanopipettes.

## 1. Introduction

The intracellular pH (pHi) is closely related to cell physiological and pathological activities, as many changes in cells are verifiably accompanied by significant changes in pHi. pHi can directly or indirectly affect cell proliferation [1], migration [2], apoptosis [3], protein processing [4], endocytosis [5], and multi-drug resistance [6]. At the same time, tissue ischemia–reperfusion and muscle contraction are also related to pHi [7]. In addition, abnormal pH is one of the important factors in the occurrence and development of diseases, including cardiopulmonary diseases, neurodegenerative diseases, cancer, Alzheimer’s disease, and so on [1,8]. Therefore, the development of well-designed pH sensors is vital for comprehending the array of physiological and pathological processes in cells. Currently, a variety of advanced methodologies, such as nuclear magnetic resonance [9,10], fluorescence spectroscopy [11], surface-enhanced Raman scattering [12], electrochemical sensing [13,14], and others, are being used for detecting pHi. Among them, electrochemical sensing, which utilizes microelectrodes, has unique advantages, including an excellent signal-to-noise ratio, rapid mass transfer rate, high steady-state current density, and minimal time constant [15,16].

The first application of a glass micropipette for pHi measurement was documented in 1975 within Purkinje fibers [17]. Since then, micropipettes have been widely used as effective sensing tools for detection and analysis [18,19]. Later, nanopipettes with smaller tips improved the spatial resolution while minimizing invasiveness, becoming a more powerful tool for detection and analysis [20,21,22,23,24,25,26]. Nowadays, various methods have been developed to prepare pH-responsive nanopipettes, among which the pH response based on ion current rectification (ICR) is a research hotspot [27,28,29,30,31,32,33,34,35]. The discovery of ICR in nanopipettes within a microfluidic environment by Bard [28] prompted researchers to realize that the intensity and direction of ICR depend on many factors, including the solution pH, electrolyte concentration, and charge density of the inner walls [27], offering novel approaches to develop pH sensors.

Introducing pH-sensitive materials onto the inner walls of nanopipettes is a useful method to improve the pH response of nanopipettes [29]. Researchers have employed various pH-sensitive materials, including inorganic substances [29,30,35], bioactive substances [31,32], and pH-responsive groups [33], to modify the inner walls of the nanopipettes individually or in combination [34]. The anticipated pH-ICR response of nanopipettes can be determined by observing the pH-dependent charge behavior displayed by these materials [36]. Physical adsorption and chemical modification are two common methods employed to modify nanopipettes. Materials such as chitosan are often modified onto the inner walls of nanopipettes through physical adsorption or similar means [29,30,35]. Though there is no denying that physical adsorption is a simple and easy way to modify nanopipettes, it lacks specificity and suffers from leakage. Chemical modification, which usually employs covalent attachment, offers a stronger chemical bond and provides specific sites, making it a more suitable method for the modification of nanopipettes. Previous studies have mostly focused on improving the pH response sensitivity performance of nanopipettes via modification while ignoring their selectivity [29,30,31,32,33,34,35]. Selectivity is another crucial feature that ensures the accuracy and reliability of pH detection in complex environments. In addition, the effect of inorganic materials on the pH response of nanopipettes has been extensively studied [29,30,35], while research on natural biomolecules associated with rich pH-sensitive groups as pH-sensitive elements remains limited [32]. So, the selection of versatile biomolecules to modify the inner walls of nanopipettes in order to prepare pH sensors with excellent pH response behavior holds considerable significance.

**Figure 1:**
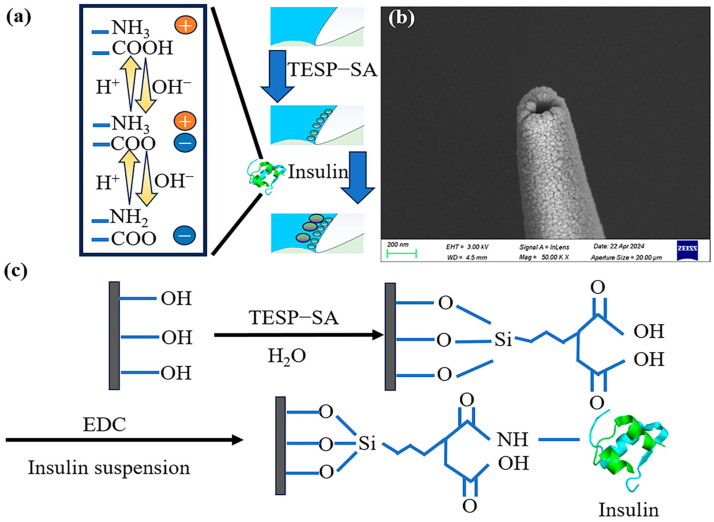
(**a**) pH response mechanism and preparation processes of insulin-modified nanopipettes. (**b**) SEM image of the nanopipettes. (**c**) Silanization and amidation of insulin-modified nanopipettes.

As we all know, insulin is a hormone that can be used to reduce blood glucose concentration and has important physiological significance. When viewed from the angle of a biomaterial, it can have even more novel applications. Bovine insulin is composed of 51 amino acids, carrying 9 amino and 13 carboxyl groups [37,38]. Amino and carboxyl groups can be used as the main connecting sites to fix insulin to the inner walls of nanopipettes through an amidation reaction with EDC as a catalyst [31]. These groups can also be used as pH response sites for pH detection (Figure 1a). Under the influence of pH, they can be reversibly bound with protons to change the charge of the inner walls of nanopipettes, thus changing the ICR intensity and the direction of nanopipettes. Then, the highly sensitive ICR response to pH is expected to be achieved. At the same time, the isoelectric point (pI) of insulin is 5.30, showing electronegativity in the cytoplasmic pH environment (7.0–7.4), and it does not have strong charge adsorption properties for ions, molecules, and bioactive substances carrying negative charges in the cells. Conclusively, insulin is anticipated to maintain its structural and electrochemical stability during testing, thereby enabling nanopipettes to exhibit selective responsiveness to pH.

In this study, biomolecular bovine insulin was employed to modify the inner walls of nanopipettes via silanization and amidation and to realize the enhanced sensitivity, high selectivity, and good reversibility pH response of the nanopipettes (Figure 1a). We monitored reaction processes and discussed the mechanism of molecules influencing the pH-ICR response behavior. This method of preparing nanosensors with excellent pH response performance by means of introducing versatile molecules onto the inner walls of nanopipettes was of great significance for promoting the practical application of nanopipettes in sensing and detection.

## 2. Materials and Methods

### 2.1. Materials and Apparatus

Chemical reagents such as ATP, GSH, and Glu were procured from Sinopharm in Shanghai, China, while 3-(triethoxysilyl)propylsuccinic anhydride (TESP-SA), 1-ethyl-3-(3dimethylaminopropyl)carbodiimide hydrochloride (EDC), 4-Morpholineethanesulfonic acid (MES), and bovine insulin were obtained from Aladdin in Shanghai, China. Different pH values ranging from 2.50 to 7.80 in 10 mM of phosphate-buffered saline (PBS) were achieved by adjusting the ratio of H_3_PO_4_ to K_2_HPO_4_ during preparation. The pH values were measured with the FE 20 pH meter made by Mettler Toledo in Zurich, Switzerland. The SEM images of the nanopipettes were captured using a Gemini SEM 300 (ZEISS, Oberkochen, Germany), operating at an accelerating voltage of 3.0 kV and coated with Au. Linear sweep voltammetry experiments were carried out using a CHI 830D electrochemical workstation from CH Instrument Co., Ltd. in Shanghai, China. BF100-78-15 borosilicate capillaries with an outer diameter of 1 mm and inner diameter of 0.78 mm were acquired from Sutter Instruments Co., Novato, CA, USA. These capillaries were used to create nanopipettes using a P-2000 laser puller (Sutter Instruments Co., Novato, CA, USA).

### 2.2. Preparation and Modification of Nanopipettes

The borosilicate capillaries were first cleaned by soaking them in freshly prepared Piranha solution at room temperature for 0.5 h and then extensively rinsed with ultrapure water. Subsequently, the capillaries were dried at 60 °C before being pulled into nanopipettes with a diameter of around 130 nm (Figure 1b) using the following parameters Heat = 350, Fil = 0, Vel = 20, Del = 145, and Pull = 180. Then, the nanopipettes were silanized by filling them with a solution containing TESP-SA (1% *v*/*v*) in ethanol and incubating at room temperature for 1 h. The nanopipettes were drained thoroughly with ethanol and then filled with MES solution (0.1 M) containing EDC (10 mg/mL) for 1 h.

The EDC was used to mediate the amide reaction between carboxyl groups on TESP-SA and amino groups on insulin. Bovine insulin was introduced onto the nanopipettes by backfilling with a mixture of 1 mg/mL bovine insulin suspension and PBS buffer (pH 7.60) containing 10 mg/mL EDC in a 1:1 ratio (*v*/*v*). The reaction was allowed to proceed overnight before washing the nanopipettes with ultrapure water and readiness for use. The I-V curves were tested by inserting nanopipettes, which were filled with pH 6.86 phosphate-removed PBS, into different solutions according to experimental requirements.

### 2.3. Electrochemical Characterization

The solutions inside and outside the nanopipettes were each inserted with Ag/AgCl wires measuring 0.3 mm in diameter. The Ag/AgCl wire within the nanopipettes served as the working electrode for recording ion currents within a potential range of −2 to +2 V at a scan rate of 0.1 V/s. The rectification status of the nanopipettes was represented by the rectification factor *r (r = log_2_ |I_+2V_/I_−2V_|)* [31], which was the logarithm processed rectification ratio (RR). It made the data more explicit and could better show the state of the inner walls of the nanopipettes. The absolute value of *r* represented the intensity of rectification, and the larger the value, the stronger the rectification intensity. When *r* was positive, the positively charged inner walls of the nanopipettes exhibited anion-selective behavior. When *r* was negative, the negatively charged inner walls of the nanopipettes exhibited cation-selective behavior. The *r* with opposite sign and the same absolute value represented the ICR with opposite direction and the same intensity.

## 3. Results

### 3.1. Optimization of Insulin Introduction Conditions

**Figure 2:**
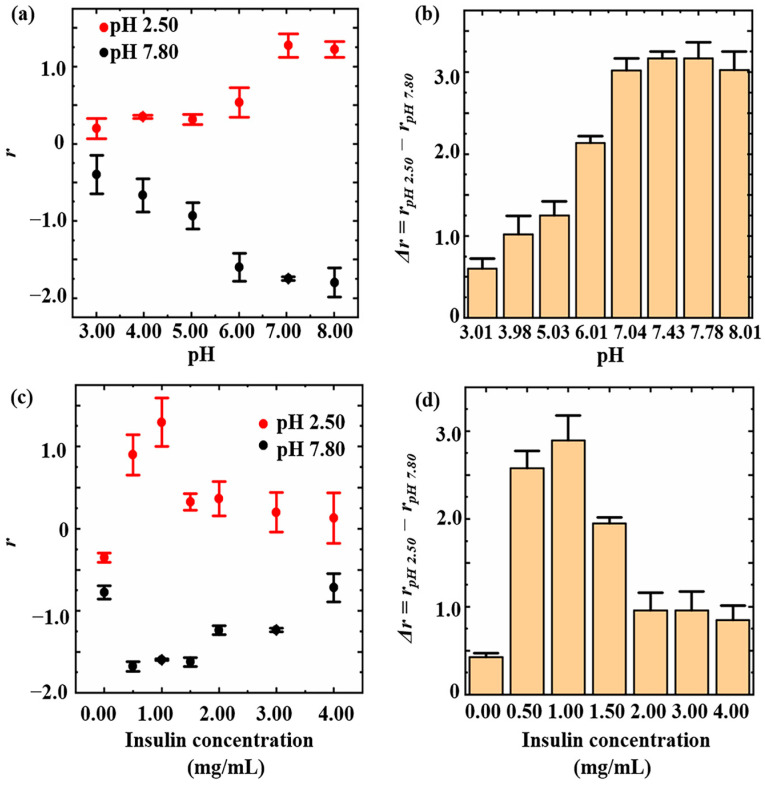
Impact of reaction solution pH on the nanopipettes’ response to pH (**a**) and their sensitivity to pH response (**b**). Impact of insulin concentration on the nanopipettes’ response to pH (**c**) and their sensitivity to pH response (**d**).

Insulin was introduced onto the inner walls of nanopipettes by a two-step reaction of silanization and amidation. Specifically, carboxyl groups were introduced onto the inner walls of nanopipettes through silanization, followed by the introduction of bovine insulin via amidation (Figure 1c). The form of insulin homo-oligomers was primarily influenced by the solution pH and insulin concentration [39], which may affect the introduction of insulin onto the inner walls of the nanopipettes. Thus, in order to achieve the optimal introduction effect of insulin onto the inner walls of the nanopipettes, the effects of the reaction solution pH and insulin concentration on the efficiency of insulin introduction were documented. From pH 3.01 to 7.04, the intensity of ICR to acid and base increased with the increasing pH values of the reaction solutions and remained at a high level from pH 7.04 to 8.01 (Figure 2a). For a more intuitive representation of the introduction efficiency, we defined *Δr = r_pH_ _2.50_ − r_pH_ _7.80_*. The higher the *Δr* value, the more efficient the introduction efficiency. When the pH value of the reaction solution increased from 3.01 to 7.04, *Δr* increased from 0.60 ± 0.12 to 3.02 ± 0.23 (Figure 2b). In contrast, *Δr* did not exhibit significant changes, remaining consistently high when the pH of the reaction solution increased from 7.04 to 8.01. This may be related to the influence of the pH on the efficiency of amidation. In EDC-mediated protein conjugations, a pH of approximately 7.5 is customarily employed to optimize amidation, which is consistent with our experimental observations [40,41]. In Figure 2c, ICR intensity varied with insulin concentration both in acid and base solutions. Specifically, increasing the insulin concentration led to increased ICR intensity when the insulin concentration was below 1.00 mg/mL. However, when the insulin concentration was higher than 1.00 mg/mL, increasing the insulin concentration led to a decreased ICR intensity. Within the range of 0.00–1.00 mg/mL, there was a gradual increase in *Δr*, ranging from 0.42 ± 0.05 to 2.89 ± 0.29 with an increasing insulin concentration. However, within the range of 1.00–4.00 mg/mL, there was a decrease in *Δr* from 2.89 ± 0.29 to 0.85 ± 0.16. This decrease in *Δr* may be attributed to the self-binding and aggregation of insulin in high-concentration [39,42], which prevents the effective introduction onto inner walls of nanopipettes (Figure 2d). Therefore, for this experiment, the bovine insulin suspension with a concentration of 1.00 mg/mL and pH value of 7.60 was used to prepare insulin-modified nanopipettes for its good pH-responsive behavior.

### 3.2. Monitoring of Modification Process

**Figure 3:**
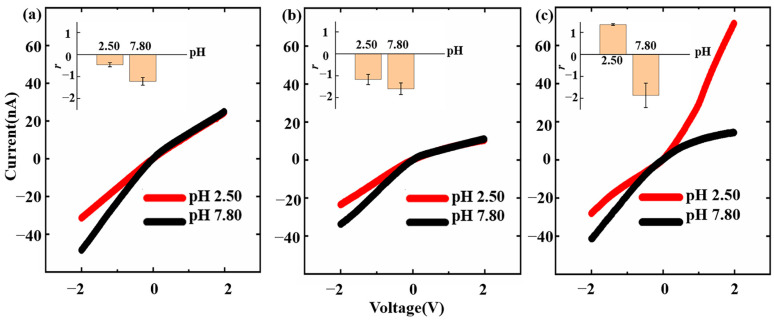
I-V curves and *r* values of (**a**) bare, (**b**) TESP-SA-modified, and (**c**) insulin-modified nanopipettes in PBS solutions at pH 2.50 and pH 7.80, respectively.

To study how modified molecules affect the ICR of nanopipettes, I-V curves were measured for bare nanopipettes, nanopipettes modified with TESP-SA, and nanopipettes modified with insulin in PBS solutions at pH 2.50 and 7.80, respectively. Figure 3a illustrates that the *r_pH_ _2.50_* and *r_pH 7.80_* values of bare nanopipettes were −0.46 ± 0.10 and −1.21 ± 0.17. This indicated that the inner walls of the bare nanopipettes were negatively charged at both pH 2.50 and pH 7.80. Previous research has shown that the separation of terminal silica hydroxyl groups on the inner walls could lead to a negative charge inside the nanopipettes [34]. Our experimental results coincided with this phenomenon and demonstrated the usability of our prepared bare nanopipettes. The negative charges on the inner walls made the bare nanopipettes exhibit cation-selective behavior, and therefore, the current under positive voltage is smaller than that under negative voltage of the same magnitude. The enhanced ICR behavior observed in the alkaline solution (pH 7.80) can be attributed to the increased negative charges resulting from the dissociation of terminal silica hydroxyl groups facilitated by the alkaline environment [31]. Moreover, carboxyl groups were introduced onto the glass inner walls of the nanopipettes through silanization using TESP-SA. Figure 3b illustrates that the *r_pH 2.50_* and *r_pH 7.80_* values of TESP-SA modified nanopipettes were −1.17 ± 0.24 and −1.60 ± 0.26, respectively, which exhibited the same rectification direction and greater intensity compared with the bare nanopipettes. The results showed that after silanization, the negative charges on the inner walls of the nanopipettes had increased, and the nanopipettes showed stronger cation-selective behavior. This verified the fact that carboxyl groups had already modified onto the inner walls because carboxyl groups were able to dissociate more readily than the terminal silic hydroxyl groups in order to introduce more negative charges.

Finally, insulin was modified onto the nanopipettes through the amidation of the carboxyl group and insulin. Figure 3c illustrates that the *r_pH 2.50_* and *r_pH 7.80_* values of insulin-modified nanopipettes were 1.36 ± 0.04 and −1.86 ± 0.55, respectively. We found that the *r* of insulin-modified nanopipettes displayed different signs in acidic (pH 2.50) and alkaline (pH 7.80) environments, indicating that the rectification direction of insulin-modified nanopipettes changed with pH. When the pH to be detected was below the pI (pI = 5.30) of insulin, insulin would carry positive charges, and the inner walls of the nanopipettes acquired positive charges. Under the influence of positive charges, the inner walls of the nanopipettes exhibited anion-selective behavior. Similarly, insulin carried negative charges when the pH to be measured was higher than the pI of insulin. Under the influence of negative charges, the inner walls of the nanopipettes exhibited cation-selective behavior. Thus, insulin-modified nanopipettes exhibited a reversed ICR direction. The reversed ICR direction allowed us to more intuitively feel the pH-induced charge change on the inner walls of the nanopipettes. In terms of the values of *r*, upon changing the solution’s pH from 2.50 to 7.80, the pH response sensitivity of insulin-modified nanopipettes (*Δr = r_pH 2.50_ − r_pH 7.80_* = 3.22) was found to be significantly enhanced compared with the bare ones (*Δr* = 0.75), with an improvement factor of 4.29.

### 3.3. pH Response Behavior

**Figure 4:**
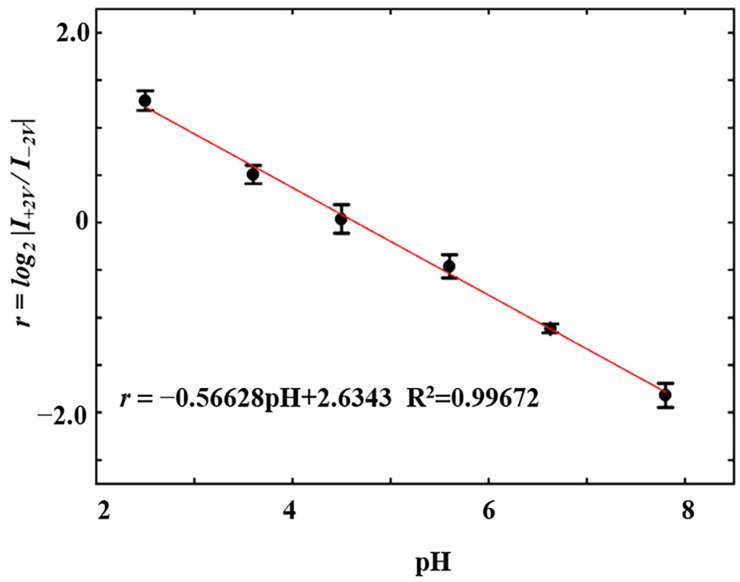
Sensitivity of the pH response exhibited by insulin-modified nanopipettes (*n* = 6).

A comprehensive evaluation was conducted on the pH response behavior of nanopipettes in terms of sensitivity, selectivity, and reversibility. The pH response sensitivity of insulin-modified nanopipettes was tested using a series of values of PBS buffers (2.50, 3.63, 4.50, 5.61, 6.63, and 7.80, which covered the range of pH changes in cells under physiological and pathological states). The results demonstrated a gradual decrease in the *r* values ranging from 1.28 ± 0.10 to −1.82 ± 0.13 as the pH values increased from 2.50 to 7.80 (Figure 4). When the pH values of PBS buffers were 2.50, 3.63, and 4.50, the *r* values were 1.28 ± 0.10, 0.51 ± 0.10, and 0.04 ± 0.15, respectively. This indicated that at these pH, the inner walls of the nanopipettes were positively charged and exhibited anion-selective behavior. When the pH of PBS buffers were 5.61, 6.63, and 7.80, the *r* values were −0.46 ± 0.12, −1.11 ± 0.05, and −1.82 ± 0.13, respectively. This indicated that at these pH values, the inner walls of the nanopipettes were negatively charged and exhibited cation-selective behavior. Linear fitting analysis revealed a strong linear relationship between *r* and pH (*r* = −0.56628 pH + 2.6343, R^2^ = 0.99672). Data fitting showed that when the pH was 4.65, the *r* was 0, and the net charge on the inner walls of the nanopipettes was zero. This pH value was marginally below the pI of insulin (pI = 5.30). The discrepancy might arise from unreacted silanol groups and carboxyl groups which introduced additional negative charges onto the inner walls.

**Figure 5:**
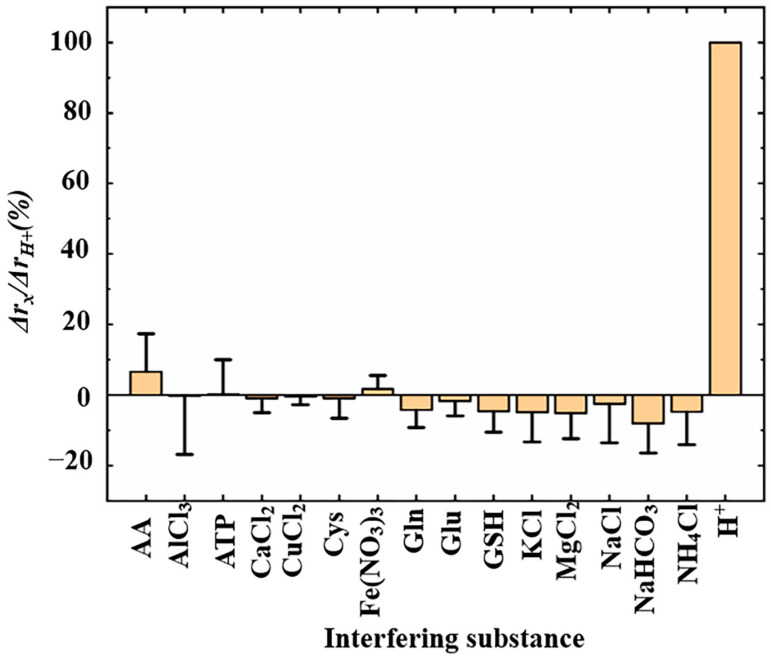
Selectivity of the pH response exhibited by insulin-modified nanopipettes (*n* = 6).

The selectivity of insulin-modified nanopipettes was achieved by adding 50 uM/L of interfering substances, which were often added in the evaluation of sensor selectivity [12,43,44], including inorganic salts (CaCl_2_, CuCl_2_, Fe (NO_3_)_3_, KCl, MgCl_2_, NaCl, NaHCO_3_, NH_4_Cl, and AlCl_3_) and small biological molecules (AA, ATP, Gln, Glu, GSH, and Cys) to PBS solution at pH 7.00, respectively. This pH value is the closest to that in the physiological state of the cells and is less able to cause the precipitation and denaturation of interfering substances. In Figure 5, we analyze data from six nanopipettes. For one nanopipette, we defined the change in ICR after the addition of 50 uM/L of H^+^ as *Δr_H+_ (Δr_H+_ = r_H+_ − r_pH_ _7.00_)*, whose value was fixed at 100% and defined the change in ICR after the addition of 50 uM/L of specific interfering substance as *Δr_x_ (Δr_x_ = r_x_ − r_pH_ _7.00_)*. The intensity of the interfering signal was normalized by *Δr_x_/Δr_H+_ (%)*. The results demonstrated that AA, ATP, and Fe(NO_3_)_3_ slightly enhanced the ICR intensity of insulin-modified nanopipettes, while CaCl_2_, CuCl_2_, Fe(NO_3_)_3_, KC1, MgCl_2_, NaCl, NaHCO_3_, NH_4_Cl, Gln, Glu, GSH, and Cys weakly reduced the ICR intensity of insulin-modified nanopipettes. Even the strongest interference signal generated by NaHCO_3_ was only 8.08% of the same concentration of H^+^ signal intensity. This indicated that insulin-modified nanopipettes had high selectivity.

**Figure 6:**
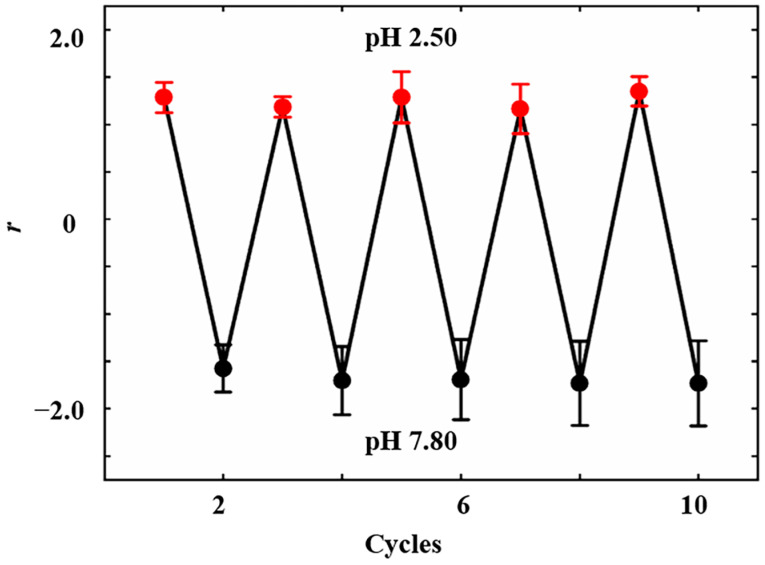
Reversibility of the pH response exhibited by insulin-modified nanopipettes (*n* = 6).

The reversibility of insulin-modified nanopipettes was tested by alternately inserting nanopipettes into solutions with pH values of 2.50 and 7.80 ten times. The results demonstrated that the ICR behavior of insulin-modified nanopipettes exhibited reversible switching between anion-selective behavior and cation-selective behavior upon changing pH values of the solution (Figure 6). It was observed that the deviation in response to acid (pH 2.50) for insulin-modified nanopipettes did not exceed 7.87%, while the deviation in response to alkali (pH 7.80) did not exceed 9.86%. This result indicated the good reversibility of the pH response for insulin-modified nanopipettes.

## 4. Discussion and Conclusions

Nanopipettes have attracted wide attention due to their unique properties, offering new and powerful tools in the field of nanosensing. Modifying nanopipettes to enhance their sensing performance is crucial. In this study, we used natural biomolecular insulin to modify the inner walls of nanopipettes, which significantly improved the pH response ability of nanopipettes. Insulin was introduced onto the inner walls of nanopipettes via a two-step reaction of silanization and amidation. The pH value of the solution and the insulin concentration during the reaction process were investigated to obtain the optimal reaction conditions for insulin introduction. Due to the limitations of material morphology, it is not possible to directly detect the functional groups and charged state on the inner walls of the nanopipettes. The experiment used I-V curves to determine the charged state of the inner walls of the nanopipettes and further determined whether the designed molecules were successfully introduced by measuring the charged state of the inner walls at different pH values. This is a method of inferring material states by characterizing material properties. In addition to electrochemical methods, some methods in acoustics [45] and electromagnetism [46,47] can also be used to inversely infer material states. The pH responsiveness of insulin-modified nanopipettes was comprehensively evaluated for sensitivity, selectivity, and reversibility.

Compared with other reports on enhancing the pH response of nanopipettes through modification, employing insulin as a functional molecule is original. Insulin-modified nanopipettes exhibited approximately 4.29 times higher sensitivity to pH response compared with bare nanopipettes. Additionally, insulin-modified nanopipettes demonstrated good reversibility and selectivity toward the pH response. While numerous synthetic molecules have achieved notable improvements in pH sensitivity when employed as functional materials on nanopipettes, the issue of pH response selectivity remains a formidable challenge. Biomolecules, which are produced by organisms and characterized by their stable presence in biological systems, may improve the selectivity and biocompatibility of sensors when used as functional materials for sensors.

However, it is essential to acknowledge that due to the limitations of our experimental conditions, this study was limited to electrochemical analysis and was absent of actual cell testing. In the development of nanopipette sensors, the pursuit persists for versatile functionalization materials, the establishment of novel sensing strategies, and the construction of sensors better suited for intracellular sensing—all of which remain key objectives for researchers in the field.

## Data Availability

Data are contained within the article and Supplementary Material.

## Supplementary Materials

The following supporting information can be downloaded at: https://www.mdpi.com/article/10.3390/s24134264/s1. Supplementary material is our original data.
